# Using focus groups to understand causes for morale decline after introducing change in an IM residency program

**DOI:** 10.1186/1472-6920-14-132

**Published:** 2014-07-03

**Authors:** Lloyd Rucker, Johanna Shapiro, Cliff Fornwalt, Keenu Hundal, Swapna Reddy, Zarema Singson, Khanh Trieu

**Affiliations:** 1 Department of Medicine, University of California, Irvine School of Medicine, Irvine, CA, USA; 2 Department of Family Medicine, University of California, Irvine School of Medicine, 101 The City Drive South, Rte 81, Bldg 200, Ste 835, Orange, CA 92868, USA; 3 Department of Medicine, University of California, Irvine School of Medicine; VA Long Beach Healthcare System, Long Beach, CA, USA; 4 Providence Health and Services, Portland, Oregon, USA; 5 The Oregon Clinic, Portland, Oregon, USA; 6 Gastroenterology Consultants of San Antonio, San Antonio, Texas, USA; 7 Southern California Kaiser Permanente Medical Group, Irvine, CA, USA

**Keywords:** Education medical graduate, Residency program evaluation, Focus groups, Qualitative research, Morale, Burnout

## Abstract

**Background:**

Although program evaluation is a core requirement of Internal Medicine residencies, little is reported in the literature regarding resident satisfaction with training. Most program evaluation consists of numerical rating scales from which it is often difficult to pinpoint exact sources of dissatisfaction.

**Methods:**

Our goal in this work is to evaluate the utility of focus group methodology to uncover in detail the reasons for residents’ deteriorating morale in an IM residency program, as well as to solicit suggestions for correction. This study employed focus groups (FG) in a qualitative research design, in which descriptive statistics from a resident program evaluation survey served to guide an intensive focus group process. Participants were 40 of 45 2^nd^ and 3^rd^ year internal medicine residents enrolled in the IM residency training program. Five chief residents were trained to conduct 5 focus groups with 8 residents in each group. The focus groups examined possible issues contributing to the deterioration of morale noted in the quantitative survey.

**Results:**

Many unexpected themes were uncovered by the FGs. Residents identified the following factors as the major contributors to deteriorating morale: 1) Pace of change 2) Process of change 3) The role of chief residents in change 4) Fear of intimidation and retaliation. Groups also suggested practical recommendations for improving the culture of the residency.

**Conclusion:**

Introducing change in residency training is a challenging process. Respectful attention to resident frustrations and solution-focused discussions are necessary to understand and improve morale. Focus groups proved to be a useful tool in revealing the precise source of pervasive resident concerns as well as providing potential solutions. In addition, FGs methodology can be adapted in a practical manner to residency evaluation.

## Background

Program evaluation is a core requirement of residency training programs [1]. Most programs utilize questionnaires with Likert ranking of answers. Some offer the option of brief written comments. Program evaluation is intended to support the goal of the Alliance for Academic Internal Medicine Education Redesign Task Force to make residency training more "resident-centered [2]". This is an important aim, as the literature documents widespread stress [3-5] and burnout [6-8] during residency. Most studies note work environment (program organization, interpersonal relationships, time pressures, and lack of control) and situational stressors, such as internal programmatic conflicts, as factors contributing to burn-out [9,10]. To identify systemic problems that result in declines in morale, a program must comprehend in depth the issues underpinning specific numerical shifts. Yet the quantitative nature of the typical survey tool precludes such deep understanding of residents’ concerns.

The study was triggered by an annual written resident survey that uncovered new resident dissatisfaction and a lower overall level of morale compared to prior years. Specifically, resident evaluations of program leadership, fairness of evaluations, and educational quality declined compared to the two previous years, as did resident personal and professional morale (see Table 1). Especially troubling was that less than two-thirds of the residents agreed that they would choose this program again, compared to 91.0% in the previous year. However, because we could not determine from our standard survey the specific nature and sources of this dissatisfaction, we conducted a series of focus groups (FGs) to gain a more explicit understanding of residents’ concerns. Focus groups are often used to gather more in-depth information about survey results; and to explore questions where differences of opinion might exist [11]. Although at first glance FGs might not seem ideally suited to the exploration of sensitive topics, in fact research suggests that they can reveal data that other methodologies do not [12].

**Table 1:** Four years of selected survey results

		**YR 1**	**YR 2**	YR 3*	**YR 4**
Quality	Leadership	3.74	3.84	**3.62**	3.73
	Fair Evaluation	3.80	3.86	**3.62**	3.90
	Educational Quality	3.99	4.11	**3.71**	4.04
Personal morale	Depressed (% total)	4.5	2.50	**3.40**	2.70
	Low	26.9	22.8	**36.4**	29.3
	Okay	35.8	46.8	**39.8**	42.3
	Good	29.9	21.5	**20.5**	25.7
Program morale	Depressed (% total)	0.0	0.0	**1.10**	0.0
	Poor	11.9	6.4	**20.5**	9.2
	Expected	70.1	66.7	**67.0**	73.2
	Better than expected	14.9	24.4	**8.00**	17.6
Choose again	Yes (%)	77.3	91.0	**62.8**	82.3
	No	3.0	1.30	**8.10**	4.1
	Unsure	19.7	7.70	**29.1**	13.6

*Year 3 was the year of concern evaluated by the focus groups.

## Methods

### Participants

Participants in the FG process were 40 of the 45 available residents from the final 2 years of training in a public university internal medicine residency program. We excluded interns because interns had not participated in the evaluation survey. Residents were free to decline, but almost all agreed.

### FG design, training, and implementation

The Program Director (PD) developed a preliminary version of the FG questions based on data from the year-end survey. The 5 chief residents and a psychologist with many years’ experience designing FG protocols and facilitating focus groups reviewed and modified this question protocol. The questions were open-ended to allow diverse opinions to emerge. The protocol included probes and follow-up questions [13].

We employed a modified FG methodology, following adjustments recommended for conducting focus groups in small communities with a high degree of social familiarity [14]. As in a small community, group participants in this study knew each other and the facilitators were "insiders," chief residents in the program, in contrast to the usual practice where group members are not previously acquainted and facilitators have no connection with participants [15]. We decided to use chiefs as the facilitators for both pragmatic and theoretical reasons. Practically, we did not have funding to hire external facilitators. We also wanted to employ a methodology that could realistically be replicated in other residencies, which similar to our program would likely not have the resources to pay for outside facilitators. From a theoretical perspective, guidelines for use of focus groups in community research encourage training trusted community members as facilitators, rather than using expert outsiders [14]. We reasoned that participating residents would be more comfortable in discussing morale issues with known individuals in whom they had respect and confidence.

We recruited 5 separate groups of residents with 8 members in each group. Each group held two sessions with a team of two program chief residents, one of whom served as facilitator, the other as note-taker.

The 5 chiefs were trained as moderators [16] in a session that included discussion, role playing, and supplementary reading materials on FG philosophy and methodology [17]. In the actual FG sessions, the chiefs made clear to participants that, as moderators, they were there to facilitate, not judge. These chiefs were new to the job and had been residents, not faculty, during the prior year when the morale decline originated. Anonymity and confidentiality for participants was ensured to establish an atmosphere of safety. All participants were aware identifying information would be redacted. At the end of each FG, the moderator summarized the main points of the discussion and asked group participants to confirm and/or modify this summary [18,19]. All sessions were audio recorded and transcribed.

### Data analysis

An audit trail of transcriptions, field notes, focus group summaries and interpretations was established [20,21]. A qualitative method known as immersion-crystallization guided data analysis [22]. In the initial phase of this process, individual researchers noted key words, phrases, and major themes [18,23]. Transcripts were reviewed by multiple members of the team [24]. Researchers then met to discuss their interpretations. In order to detect core patterns and comments, we evaluated the discussion for the frequency of themes and the intensity of comments. Resolution of disagreements was achieved through discussion. As a result of this analytic process, the researchers consolidated their findings into the four themes reported below.

This research project was reviewed and approved by the University of California Irvine Office of Research institutional review board (HS#2011-8427). Because information was collected in a manner such that no identifiers were used and participants could not be linked to the data, the study was approved as exempt research and written consent was not required. All potential participants were given information about the goals and methods of the study. They were informed that they were being asked to take part in research; what their participation would involve, including risks and benefits; and how their privacy and confidentiality would be protected. Because participants were residents in the program, we emphasized that participation was voluntary, and that participants could withdraw at any time or refuse to participate without any adverse consequences. Everyone who agreed to take part in the study consented verbally to their involvement.

The reporting of this study conforms to RATS guidelines for qualitative research. In particular, it presents an argument for the relevance of the study question. It provides explanation and justification for the particular qualitative methodology selected. It demonstrates transparency in describing study procedures, including sampling methods and recruitment strategies, data collection and analysis, roles of researchers and ways in which possible conflicts of interest were addressed. It discusses informed consent and efforts to guarantee anonymity and confidentiality to study participants. Finally, it attempted to draw conclusions based in the literature that did not overreach and that honestly recognized study limitations.

## Results and discussion

There was widespread agreement across all groups that morale had declined. Researchers identified 4 main themes contributing to this demoralization: the pace of change; the process by which change was introduced; alteration in the role of chiefs from resident advocates to agents of change; and fear of retaliation. Each theme contained a number of "surprises" that had not been expected by the PD or the residency program, and could not have been deduced from the survey questionnaire results.

### Theme 1: the pace of programmatic change

Program changes included a shift away from float night coverage to overnight call; the introduction of a new morning conference; and the implementation of a weekly protected half-day of focused educational experience (the Academy). FG participants attributed some of the deterioration of morale to the many changes that had occurred over a short period of time, leaving them feeling helpless and out of control. In the words of one resident, "It has been this culture over the last twelve months of constant change… it felt like a completely different residency program" (Group 4). In itself, this theme was self-evident, as multiple, simultaneous changes are obviously stressful. However, a revelation to administration was that residents found even positive changes such as new educational conferences to be nerve-racking, principally because of the time commitments involved. "Even some of the things which I think are good, adding the academy, morning report, caused some stress last year because they were new and they changed the routine" (Group 3). A minority of residents disagreed: "Bringing morning reports, introducing the academy-where you get to see your colleagues – these were great changes-everyone likes that. It’s better having it than not" (Group 4).

The depth and strength of resident feelings was also surprising. As one resident put it, "We feel like we’re guinea pigs. That we’re being experimented on, especially the call schedule" (Group 3). Many residents described intense frustration and disorientation in response to perceived loss of structure, instability, and uncertainty. "This program has changed completely… to deal with the stress of all of these changes kind of brings us down" (Group 1). "The whole experience was awful, incredibly awful, the team was completely fragmented" (Group 5). All groups reported an unexpected level of anxiety and distress. However, a few residents were more philosophical: "Big changes take awhile for people to adjust to; people aren’t going to like it in the beginning" (Group 5). Another disputed the negative effects entirely: "The environment is excellent overall, I don’t feel overworked, and there is agood balance of teaching and time off" (Group 4).

### Theme 2: the process of change

According to participants, the process by which change was introduced was more stressful than the changes. This too was an unanticipated outcome for the residency program which, in its own eyes, had made every effort to take account of resident perspectives and include residents in all decision making processes. Still, many residents did not feel that they had a say in the call schedule change – to them, it seemed superimposed from above. "It seemed like there was some sort of charade to make it look like this was what residents wanted" (Group 2). In their view, resident feedback sessions were not really relevant to the actual change process. "Whether or not the vote actually counted for anything I’m not sure and I actually doubt it" (Group 1). The process "seems like it was a farce" (Group 4). "People ended up being insulted by the whole process. Why bother telling us you want our opinion when you’re just going to do it anyway?" (Group 4).

### Theme 3: chief residents as agents of change compromised their more important role of resident advocates

Much of the responsibility for morale decline was placed at the feet of the prior year chiefs, yet another unanticipated finding. Because of their competence and the respect in which they were held by both the residency director and their fellow residents, these chiefs were given the task of presiding over the transition. Yet, confronted with changes that were dramatic and in some cases unpopular, residents largely misconstrued this liaison role and tended to blame the chiefs for their difficulties with the new system. For example, some residents described them as agents of the administration who "…didn’t care…were more of a watchdog than helpful" (Group 1). One resident summed up the sense of vulnerability: "There was a pervasive feeling that you don’t have somebody you can tell things to, or air your complaints to, and not feel that there may be some type or retributions or that it’s not going to stay confidential" (Group 4). However, the anger at the previous chief was not universal. As one resident pointed out, "The chiefs drop in occasionally, heck to see how the teams are doing: ‘Hey guys, how’s it going?’ They bring candy. The chiefs help with how to put in orders or deal with systemic problems" (Group 2). Another liked that a chief was present at the sign-out process, "…not for sake of correcting people, but to make sure things are okay-giving specific feedback verbally, what to do if patient has this…" (Group 4).

### Theme 4: fear of intimidation or retaliation

The process of change, in turn, was affected by perceptions about the culture of the residency. A minority of residents considered the atmosphere of the program to be fairly positive, which corresponded to the perceptions of the administration. "[The PD] is receptive to hearing feedback… he’s pretty responsive" (Group 5). Others considered the program to be "somewhere in between" openness and retaliation (Group 2). A resident from a different group protested, "I do not consider the atmosphere to be at all intimidating. It’s always hard to receive feedback, and in this program people try to give feedback in a helpful manner" (Group 1).

However, the majority was more negative, stating that the program culture often was actively hostile, defensive, and resistant to feedback. Again, the intensity of resident feelings was startling to the residency program. "At residency meetings, the administration is antagonistic and intimidating to the residents" (Group 2). Residents reported feelings of being ignored, discounted, or not legitimized. They expressed concern that speaking out might jeopardize their landing future fellowships. "People are afraid for their fellowships; they’re afraid for their futures, you piss off the wrong person…" (Group 3). They appeared afraid of upsetting people in power. "When you’ve been open and been attacked, it makes you not speak up anymore" (Group 3).

Fear of retaliation existed in part because of the perceived lack of anonymity in registering complaints. Many residents were not aware that anonymous reporting mechanisms existed. It was pointed out that evaluations could easily be traced back to particular persons. "End-of-block evals are not anonymous… I made a comment about the call system. I wrote some very strong comments and I was called in for that" (Group 5). A pervasive sense of evaluations being misused in a punitive way was also uncovered. Some residents reported examples of "quid pro quo" evaluations – situations where they felt faculty retaliated against an honest evaluation with a negative evaluation of their own. "… Most people felt like they couldn’t give an honest eval because what happens is the attendings will wait and look for it" (Group 4). In a minority view, one resident offered the alternative suggestion that "Maybe chiefs reported things out of concern for the person. And you could think about being called in to the PD’s office as a way to offer help and show concern" (Group 2).

Overall, however, across all five focus groups, in the opinion of the research team analyzing the transcripts, the intensity of the language used conveyed a sense of moral outrage and bitterness that could not have been predicted from the survey results alone. Moral outrage is defined in the social sciences as a response to transgressions by others based on individuals’ expectations of perceived rights and privileges. Moral outrage is an outgrowth of moral distress, and occurs when distress cannot be relieved [25]. The residents’ sense of immunity from certain behaviors they perceived to be wrong seemed to be repeatedly violated according to their subjective judgments. The results of this demoralization were significant. Several residents said they wouldn’t pick this program again. Another added, "I can’t wait until it’s done" (Group 3) while someone else chimed in, "I’ll never come back here again" (Group 3).

As a result of the FG process, we developed crucial insights into what had gone wrong with a process of change that the administration believed to be inclusive and transparent. While cautious of generalizing from the experience of a single program, we believe there are relevant conclusions other training programs should consider.

First, because residents are stressed and often overwhelmed [3], the protocols and parameters of a decision-making process must be clear before that process begins. Otherwise residents may have unfulfilled expectations and feel as though their time has been wasted. Residents’ views and desires should always be treated respectfully, even when they cannot be entirely met. As other research has shown, extensive preparation and involvement of residents in change process is associated with greater satisfaction [26].

Further, change creates stress, and more change creates more stress. Residents perceived some changes as adverse while others were in concept positive and productive. However, the sheer volume of near-simultaneous changes created undue stress and affected morale. This was especially true because even changes that were perceived as positive required increased time commitment from the residents. The FG process uncovered dissatisfaction with increased educational time and resources, a surprising phenomenon which was not at all evident in the paper survey where these new conferences were given high marks.

As well, residents can feel intimidated, powerless, and vulnerable to abuse [5]. The PD, chief residents, and faculty underestimated the degree to which residents felt unable to openly express opinions and the difficulty of obtaining sufficient buy-in for successful change [27]. The PD believed that the change process had been extensive and inclusive. Nevertheless, while structures were in place for feedback and input, residents felt as though their comments or evaluations would lead to reprisals.

Many medicine residents plan to go on to additional fellowship training. Success in obtaining fellowships is highly dependent upon the recommendations of faculty. Residents seemed especially reluctant to openly express their views for fear of not being able to obtain fellowship positions. To counteract such deep-seated fears, extra care must be taken to guarantee resident safety in dialoguing openly about problems. Chief residents play a crucial mediating role between faculty and residents. Chiefs are among the best of the program’s house officers, and for this reason are asked to stay on as faculty for an extra year to teach and supervise. Both the program administration and residents regarded the chiefs who guided the change process as capable, hardworking, and respected. They were viewed as excellent teachers and innovators. For these reasons, the administration felt that they would be key persons to explain and promote change. However, while program administration felt that giving chief residents an important role in the change process would be beneficial, in fact placing the chiefs in that position led to loss of their credibility as resident advocates, a crucial aspect of the chief resident role. In the eyes of their fellow residents, once they were required to become "agents of change" for the administration, they were perceived as unsafe. Therefore, they had diminished capacity to function as effective problem-solvers and as liaison to the administration.

Although the large majority of the FGs expressed significant dissatisfaction, this was by no means a universal response. There were dissenting opinions which were more supportive of the residency, more sympathetic to the constraints on the PD, and more tolerant of the change process. Although the FG process itself did not provide specific insights regarding why certain residents seemed more positive and resilient, in discussions of the research team it appeared that these individuals seemed to have an unusually strong capacity to deal with adversity and unexpected situations. This reminds us that introducing change elicits a range of responses in residents, influenced in part by their attitudes toward their working conditions [28], and suggests the importance of channeling more positive residents as leaders in the change process.

### Limitations

This study was limited by a number of factors. The study was constrained by unavoidable modifications made in the FG methodology, especially the use of current chief residents as FG facilitators. However, these changes also made the FG process more realistic for other residency programs. The faculty researchers made every effort to ensure that the facilitators understood the importance of a neutral stance and did not have any "axe to grind" based on their own experiences in the residency. The team spent significant time in the training session practicing nonjudgmental questions and probes with the facilitators to reduce potential contamination of the findings.

Another potential limitation is that, despite conscientious efforts to give residents a choice about participating in the study, residents may have felt pressured to attend the FGs; and the possibility of coercion in turn might have inhibited the honest expression of critical viewpoints. However, based on the content of the data, we believe that the FG participants felt safe enough to express authentic opinions that accurately reflected their concerns. It is at least equally if not more likely that the high participation rate reflected the strength of resident feelings.

Finally, it is possible that our choice of methodology – focus groups rather than individual interviews – may have skewed the findings. In other words, there is a concern that a focus group, by voicing strong affect in a particular direction, may result in an overrepresentation of similar findings. An individual interview might produce more accurate feelings. The literature on focus groups addresses this point by emphasizing the importance of contrary opinions; and in its methodology, often explicitly requests opinions different from those being expressed [29]. The evidence that we succeeded overall in this regard is the existence of dissenting positive opinions.

Most qualitative researchers acknowledge that there are advantages and disadvantages to both focus groups and individual interviews. For example, focus groups are preferable in terms of obtaining a real-world response and avoiding tendency to please the interviewer. Focus groups are also considered superior when wishing to explore whether consensus or disagreement exists about an issue. On the other hand, individual interviews can better generate in-depth information about a single individual. They avoid the influence group dynamics may exert; and they are generally more useful when probing extremely sensitive subjects [30]. On balance, because we wanted to stimulate discussion among the residents and elicit multiple perspectives, we felt focus groups would better serve our purposes. Then too, as other researchers observe, they are a more efficient approach when resources are limited.

## Conclusions

The FG process helped this medicine residency program to uncover a depth of dissatisfaction not previously recognized, and furthermore, uncovered sources of dissatisfaction which were surprising and could not have been determined from the mostly Likert scale written survey. Researchers note that "critical events" in training can be a source of both distress and personal growth [31]. We hoped that, using the FG process, we could understand residents’ dissatisfaction and decreased morale in a way that would point us in the direction of programmatic improvement. In fact, understanding those issues in a multifaceted way led to the implementation of substantial programmatic changes with the goal of improving morale and wellbeing [28]. These included new mechanisms for anonymous feedback, bundling of faculty evaluations, expanded forums for communication, implementation of routine focus groups as standard in the evaluation process, chief residents being asked to play more of an advocacy role for residents rather than function as extensions of the administration, and an overall entirely different approach and sensitivity to change. Without the insights gained from the FGs, such directed and specific change would not have been possible. In a gratifying development, in the following year resident scores in the areas of morale and leadership improved (See Table 1). While we cannot claim causality or even correlation, we feel that the findings of the focus groups enabled us to quickly – but cautiously! –enhance the program in ways that benefitted residents and administration alike.

## Competing interest

The authors state that they have no conflict of interest in conducting this research.

## Authors’ contributions

LR originated the idea for the study and was responsible for its intellectual conceptualization. He also organized the research team. He was the primary developer of the question route used in the study. He played a major role in the interpretation of the data. He had a major role in conceptualizing and writing of the paper reporting study findings. JS provided expertise in focus group methodology. She provided two training sessions in FG methods for the research team; and continued to provide guidance throughout the study. She was responsible for troubleshooting problems in the design and implementation of the study and in data analysis and interpretation. She played a major role in data analysis and interpretation, as well as in preparing multiple drafts of the paper reporting study findings. HF, KH, SR, ZS, and KT participated in focus group methodology training sessions and made substantial contributions to the design and content of the question route. They all conducted focus groups and participated in the data analysis. They reviewed multiple drafts of the paper and contributed critical changes and suggestions. All authors have read and approved the final manuscript.

## Authors’ information

LR was Program Director for the UCI-SOM Internal Medicine Residency Program at the time of the study. JS is Director of the Program in Medical Humanities & Arts, UCI-SOM. HF, KH, SR, ZS, and KT were Internal Medicine residency program chief residents at the time of the study.

## Pre-publication history

The pre-publication history for this paper can be accessed here:

http://www.biomedcentral.com/1472-6920/14/132/prepub
